# *prolfquapp* — A User-Friendly Command-Line Tool Simplifying Differential Expression Analysis in Quantitative Proteomics

**DOI:** 10.1021/acs.jproteome.4c00911

**Published:** 2025-01-24

**Authors:** Witold E. Wolski, Jonas Grossmann, Leonardo Schwarz, Peter Leary, Can Türker, Paolo Nanni, Ralph Schlapbach, Christian Panse

**Affiliations:** † Functional Genomics Center Zurich (FGCZ) - University of Zurich/ETH Zurich, Winterthurerstrasse 190, CH-8057 Zurich, Switzerland; ‡ Swiss Institute of Bioinformatics (SIB) Quartier Sorge - Batiment Amphipole, 1015 Lausanne, Switzerland

**Keywords:** proteomics, statistical software, differential expression analysis, workflows

## Abstract

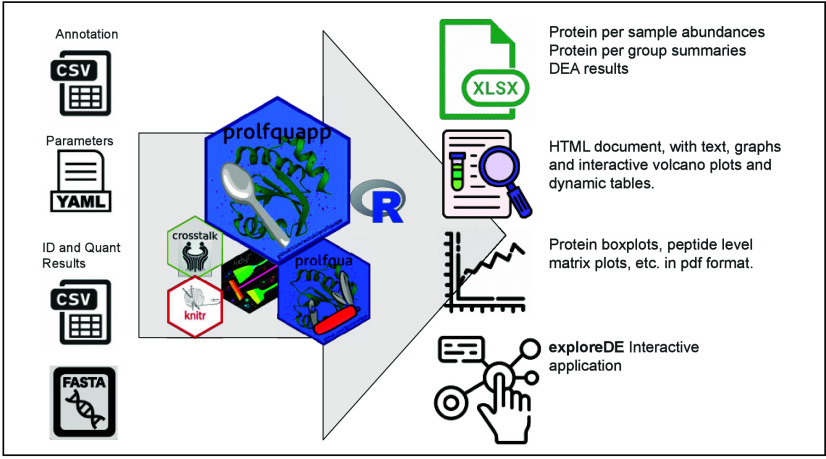

Mass spectrometry is a cornerstone of quantitative proteomics, enabling relative protein quantification and differential expression analysis (*DEA*) of proteins. As experiments grow in complexity, involving more samples, groups, and identified proteins, interactive differential expression analysis tools become impractical. The *prolfquapp* addresses this challenge by providing a command-line interface that simplifies *DEA*, making it accessible to nonprogrammers and seamlessly integrating it into workflow management systems. *Prolfquapp* streamlines data processing and result visualization by generating dynamic HTML reports that facilitate the exploration of differential expression results. These reports allow for investigating complex experiments, such as those involving repeated measurements or multiple explanatory variables. Additionally, *prolfquapp* supports various output formats, including XLSX files, *SummarizedExperiment* objects and rank files, for further interactive analysis using spreadsheet software, the *exploreDE* Shiny application, or gene set enrichment analysis software, respectively. By leveraging advanced statistical models from the *prolfqua* R package, *prolfquapp* offers a user-friendly, integrated solution for large-scale quantitative proteomics studies, combining efficient data processing with insightful, publication-ready outputs.

## Introduction

The *prolfqua* R package^1^ for differential expression analysis is becoming increasingly popular and has been used in several recently published proteomics studies.^2−5^ The *prolfquapp* package, which builds upon the *prolfqua* package, implements a ready-to-use command-line application that starts from outputs of popular quantification software,^6−8^ and allows to analyze experimental designs most commonly used for protein expression analysis.^9,10^ While the *prolfqua* package is a tool for bioinformaticians, *prolfquapp’s* primary users are researchers conducting quantitative proteomics studies.

*Prolfquapp* enables users with minimal programming experience to execute a differential expression analysis and simplifies the integration into workflow managers. The software performs protein or peptide differential expression analysis and generates dynamic HTML reports that contain quality control plots and interactive visualizations. Furthermore, we export results in multiple formats, including XLSX files, .rnk or .txt files for gene set enrichment and over-representation analysis, and the SummarizedExperiment^11^ for interactive visualization in the *exploreDE*(12) application.

Other R packages, such as *Einprot*,^13^*LFQAnalyst*,^14^*MSDap*,^15^*Amica*,^16^ or *MSstatsShiny*(17) have a similar aim, to make *DEA* analysis end user-friendly. To model the differential expression analysis or to perform the data preprocessing, these packages use methods implemented in the packages *limma*,^18^*MSstats*,^19^ or *msnbase*,^20^ while we use models and methods implemented in the package *prolfqua*.^1^ These R packages enable nonprogrammers to perform *DEA* by implementing a graphical user interface^14,17,21,22^ using *Shiny*,^23^ or implement a facade^24^ that provides a simplified interface to otherwise complex code and allows running a complex analysis with only a few lines of code.^13,15,17^ These packages, designed for interactive analysis, may not be ideal when reading the data, and model fitting takes a long time.

In the *prolfqua* publication,^1^ we discussed the advantage of modeling missing data instead of removing proteins or peptides with missing observations or imputations. There, we showed that the approaches to model missing observations, implemented in packages *prolfqua msqrob2*,^25^ or *proDA*,^26^ results in higher AUCs on all data than, for instance, *MSstats* that advocates filtering out of proteins with a large fraction of missing values and imputation. Therefore, *prolfquapp* does not offer imputation or filtering options based on missing values.

Furthermore, we aim for a minimal set of sensible input parameters and provide defaults, which will work with most data sets compatible with relative quantification approaches. These are data sets where only a small proportion of proteins exhibit changes in abundance, while the majority remain stable within a consistent protein matrix.^27−29^ As a result, the analysis typically needs to be run only once and, in rare cases, twice. The only parameters we allow easily to be changed that affect the differential expression analysis are the normalization method, with *vsn*(30,31) being the default, and turning on or off fold change inference for proteins with an excess number of missing values, with the default to model missingness. If disabled, we still report the protein without fold change and p-value. However, we do filter the data of the quantification software; for instance, we filter *DIA-NN* results by the q-value, or *FragPipe* peptide spectrum match results by peptide prophet probabilities or purity thresholds, removing all observations not meeting the filtering criteria. Here, we provide tested and recommended filtering thresholds.

We encourage and facilitate the interactive analysis and filtering of the differential expression analysis results. We not only integrated *prolfquapp* with the *exploreDE* Shiny application, but we provide summary statistics for each protein and peptide, such as the number of peptides per protein in the experiment or sample or the number of missing values per group. Based on this information, we can refine the lists of significantly differentially expressed proteins to include in the over-representation analysis (ORA) or incorporate them into gene-set enrichment analysis (GSEA). We can filter using spreadsheet software such as Excel or OpenOffice since we store the results in XLSX files. Also, when using the *exploreDE* application, which reads SummarizedExperiment (SE) output generated by the *prolfquapp*, we can filter the data by the number of peptides per protein observed in the experiment, an important criterion for the quality of the protein identification.^32^

Since in most proteomics experiments, approximately a third of proteins are identified by less than two peptides, there is concern that removing proteins or peptides from the list after the DEA analysis will lead to different results than if they had been removed beforehand. For example, some may assume that shortening the list of proteins would artificially increase the false discovery rate (FDR). However, the FDR, estimated using the Benjamini-Hochberg correction,^33^ depends primarily on the p-value distribution relative to the distribution of p-values under the null hypothesis (*H*_0_, a uniform distribution), not the absolute number of proteins tested. While methods controlling the Family-Wise Error Rate (FWER) are sensitive to the total number of tests, the FDR estimation is unaffected as long as the p-value distribution remains unchanged (see Supporting Material 1). For the GSEA analysis, we recommend using the t-statistic, which is computed independently for each protein and will not change if we remove proteins before analysis. Thus, proteins quantified by a single peptide are included by default in the analysis, and we recommend removing them, and then to compare GSEA results on the complete and filtered list. If desired, they can be removed when importing the data into *prolfquapp*.

Data management systems such as B-Fabric^34^ and OpenBIS,^35^ which schedule bioinformatics compute jobs via platforms like Galaxy^36^ and SLURM,^37^ utilizing tools such as *FragPipe* and *DIA-NN*, and enable reproducible science, are critical in modern bioinformatics. These systems automate complex analyses and manage large data sets, enhancing efficiency and scalability. We developed *prolfquapp* to integrate seamlessly with these platforms, enabling users to efficiently queue differential expression analysis (*DEA*) jobs while ensuring reproducibility across diverse computational environments. With its command-line interface, *prolfquapp* can be easily incorporated into these workflow management systems, increasing its utility for large-scale experiments where interactive analysis may be impractical. Furthermore, we wanted to enable our users, frequently using Windows computers, to replicate or modify the analysis by updating the annotation and parameters.

## Methods

### Sample Annotation

*Prolfquapp* formalizes how to annotate samples with explanatory variables for some of the most common experimental designs: parallel group design and factorial designs with and without repeated measurements. We defined an annotation file format that provides the explanatory variables and specifies the group comparisons. The first column is reserved for the sample identifier, which is either the raw file name in the case of label-free experiments or the channel for labeled experiments. The values provided in that column must match the sample names in the quantification software outputs. We support the creation of annotation files using the prolfqua_data set script which extracts the sample names used by the quantification software. The column names can be upper or lowercase. Further columns are used to assign the explanatory variables and define the contrasts to compute (see Table 1). The column *subject*, which can be used to provide, for instance, patient identifiers in paired experiments, is optional. Instead of the column *control*, the columns *ContrastName* and *Contrast* can be provided to specify the differences to compute (see Table 4).

**Table 1:** Specification of the Table We Use to Annotate the Samples and Specify the Experimental Design, as Well as Contrasts^a^

Column Name	What
relative.path/path/raw.file/channel	The sample identifier for each row must be unique
name	Sample name used in tables and figures
group/experiment	Main factor
subject/bioreplicate	Blocking factor (optional)
control	Specify the control (C) or treatment (T) condition
ContrastName	Explicitly specify the contrast name
Contrast	Specify the contrast

a You either specify the column control or the columns ContrastName and Contrast. We compare all groups to the control reference group specified in the control column. Alternatively, you explicitly specify the contrasts in the columns ContrastName and Contrast.

### Integrating Protein Information from FASTA Files

Because there are differences in what information about proteins is provided in the outputs of the quantification software, *prolfquapp* enriches the quantification data with protein information extracted directly from the FASTA file. We parse the FASTA file using the *seqinr* R package^38^ to compute protein lengths and the number of theoretical tryptic peptides, use them to compute intensity-based Absolute Quantitation (iBAQ) values, and parse the protein descriptions to extract gene names. We are adding this information to generated reports.

### Parsing of Quantification Results

Prolfquapp builds on the *prolfqua* package which stores all the data in a tidy data table containing the explanatory and response variables for all the proteins. A configuration object provides annotations of the columns in the tidy data table. We automate the process of parsing quantification results and creating a configuration for various output formats. Table 2 shows which output formats are currently supported and if protein or peptide-level *DEA* is possible.

**Table 2:** Quantification Software Supported at the Time of Publishing^a^

Software	Input file	–software
DIANN/FragPipe DIA	report.tsv	**DIANN** or **DIANN_PEPTIDE**
FragPipe DDA workflow	msstats.tsv	**MSSTATS** or **MSSTATS_PEPTIDE**
FragPipe TMT workflow	PSM.tsv	**FP_TMT** or **FP_TMT_PEPTIDE**
MaxQuant	peptide.txt	**MAXQUANT** or **MAXQUANT_PEPTIDE**
Spectronaut	BGS report	**BGS** or **BGS_PEPTIDE**

a The second column shows which file is used as input. The third column shows which parameter must be passed to the prolfqua_dea and prolfqua_qc applications to generate protein or peptide centric reports.

Quantification software outputs might vary depending on the q-value thresholds applied. Therefore, we filter the data using recommended settings or what we believe are sensible values. For instance, we filter *DIA-NN* data to protein group q-Value of 0.01, or when importing the FragPipe *psm.tsv* file, we use for TMT quantification, we filter for a purity of 0.5 or greater and peptide prophet probability greater than 0.9. It is possible to adjust these thresholds in the parameter YAML file.

Developing an application that integrates multiple upstream processing software presents challenges in handling diverse tabular file formats. These challenges go beyond column naming or format (tidy vs wide) and include differences in how quantification software extracts protein identifiers from FASTA files. Different tools may assume UniProt format and parse identifiers. However, some software tools also allow for the specification of custom parsing rules. There are also inconsistencies in how quantification software represents file names, with some tools removing extensions, others retaining them, and some even keeping the full file path, which may differ based on the operating system. Despite efforts to standardize file structures across software versions, import functions in *prolfquapp* often require updates.

Although we provide implementations of import functions for some of the popular quantification software, we make it possible to inject custom file-parsing functions from dedicated R packages if they are compatible with the *prolfquapp* parsing interface. The *prolfquappPTMreaders* package^39^ is an example of an external package that implements *prolfquapp*-compatible methods for reading outputs of FragPipe PTM workflows.

### Design and Parameter Management

We pass parameters to the programs using command-line arguments or YAML configuration files. YAML is structured, easy to read, and allows users to define key parameters, such as the data normalization method. It also includes details about the input data, project, and work units, which we integrate into the HTML reports. While this may not be crucial for single data sets, it becomes vital when managing multiple data sets or interoperating with a LIMS system.

To modularize and structure the code, we utilized R6 classes. For instance, the AnnotationProcessor class handles methods for parsing the sample annotation file, AppConfiguration manages the YAML parsing, QCGenerator contains functionality for the QC application, and DEAAnalyse organizes methods for the *DEA* application.

### Generating Interactive HTML and XLSX Documents

An important functionality of the *prolfquapp* package is to report *DEA* results. We use the R bookdown package^40^ to generate the main HTML report, which allows us to create figure and table captions and reference them in the text. We used *ggplot2*(41) and *plotly*(42) to create figures and add interactive features such as hovering and highlighting. We implement tables using the *DT* package,^43^ enabling sorting, searching, and filtering of data. The tables interact with the plots through the crosstalk^44^ package, allowing us to highlight selected items in the plots based on table selections for linked exploration. Additionally, we use the *writexl* package^45^ to generate XLSX files that include protein intensity estimates and statistical results for further analysis.

### Data Set Used for Benchmarking

To demonstrate the *prolfquapp* packages functionality we used the renal cell carcinoma data set^46^ sourced from MassIVE Reanalysis - RMSV000000696.1. This data set was produced using the FragPipe DIA workflow.^47^ The data set comprises 187 samples, and the FragPipe DIA workflow identified and quantified 8624 proteins and 94587 peptides. The key report.tsv file is approximately 18GB in size.

## Results and Discussion

### Workflow

To quantify peptidoform or protein abundances, we use protein quantification software that processes mass spectrometry raw data and FASTA files. The quantification software stores the results in tabular text files within a directory. Here we show how the *prolfquapp* command-line tools can be executed on a Linux or macOS shell to generate QC reports and perform a DE analysis (for Windows, we provide bat files).

**Figure 1:**
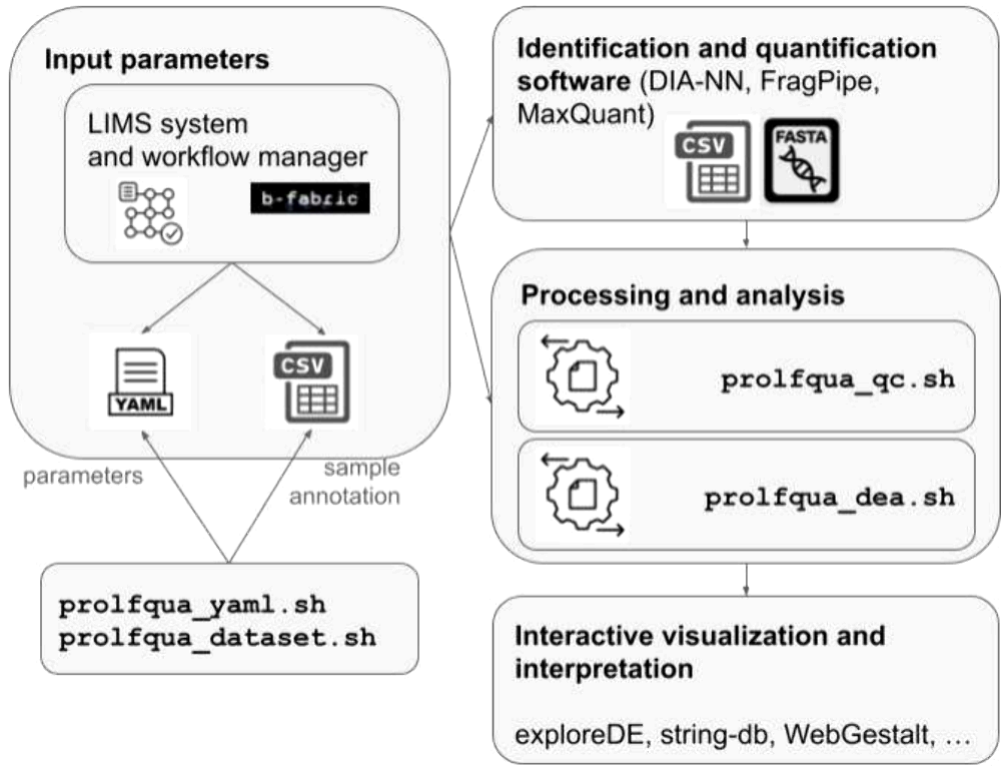
*DEA* analysis workflow using *prolfquapp*. Input parameters and sample annotation are either generated by the LIMS system or a workflow manager, or using the prolfqua_yaml and prolfqua_data set scripts. Then, the prolfqua_qc and prolfqua_dea scripts can be run to analyze the identification and quantification software outputs. We support interactive data visualization and data interpretation by generating outputs compatible with *exploreDE* and *string-db*.

The first step when modeling the peptide and protein abundances is to generate a file that annotates the samples with explanatory variables. Ideally, this information is stored in a LIMS system, and using an API, the data can be extracted and written into an annotation file (see Figure 1). In case there is no LIMS system, we can run the command:

./prolfqua_data set.sh -i data_dir/ -s DIANN -d annotation.xlsx

which will parse the quantification result from the data_dir and extract the names of all raw files (label-free) or channels (labeled quantification) and prepare a table to add sample names, the grouping variable, and contrast information (Table 1, Table 2).

The next step is to run the quality control, which generates HTML outputs that visualize the data and help to identify outlier samples or determine if samples were contaminated. Table 5 summarizes outputs generated by the QC application.

**Table 3:** Annotation File Listing a Subset of Raw Files, Sample Names, Sample Groups (Group), and Patient Identifiers (Subject)^a^

raw.file	Name	Group	Subject
CPTAC_CCRCC_W_JHU_20190112_LUMOS_C3L-00004_NAT	NAT_Old_4	NAT_Old	4
CPTAC_CCRCC_W_JHU_20190112_LUMOS_C3L-00004_T	T_Old_4	T_Old	4
CPTAC_CCRCC_W_JHU_20190112_LUMOS_C3L-00010_NAT	NAT_Young_10	NAT_Young	10
CPTAC_CCRCC_W_JHU_20190112_LUMOS_C3L-00010_T	T_Young_10	T_Young	10
CPTAC_CCRCC_W_JHU_20190112_LUMOS_C3L-00011_NAT	NAT_Old_11	NAT_Old	11
CPTAC_CCRCC_W_JHU_20190112_LUMOS_C3L-00011_T	T_Old_11	T_Old	11
CPTAC_CCRCC_W_JHU_20190112_LUMOS_C3L-00026_NAT	NAT_Old_26	NAT_Old	26
CPTAC_CCRCC_W_JHU_20190112_LUMOS_C3L-00026_T	T_Old_26	T_Old	26
CPTAC_CCRCC_W_JHU_20190112_LUMOS_C3L-00079_NAT	NAT_Young_79	NAT_Young	79

a The values from the “Name” column will be used in the report figures and tables; therefore, they should be short yet descriptive.

**Table 4:** Table Showing Two Additional Columns Used to Specify the Contrasts^a^

ContrastName	Contrast
T_vs_NAT	(G_T_Old + G_T_Young)/2 - (G_NAT_Old + G_NAT_Young)/2
Old_vs_Young	(G_NAT_Old + G_T_Old)/2 - (G_NAT_Young + G_T_Young)/2
T_vs_NAT_gv_Young	G_T_Young - G_NAT_Young
T_vs_NAT_gv_Old	G_T_Old - G_NAT_Old
DoesTvsNatDependsOnAge	T_vs_NAT_gv_Old - T_vs_NAT_gv_Young

a Please note that the group names have the prefix “G_” (shorthand for Group).

**Table 5:** List of Output Files Generated by *prolfquapp* Applications for QC and DEA (see Supporting Material 1)

Application	File name	File content
prolfqua_qc	QC_sampleSizeEstimation.html	Experiment QC and Sample Size Estimation Report
	QC_porteinAbundance.html	Interactive HTML report displaying protein iBAQ values
	proteinAbundances.xlsx	Spreadsheet with peptide and protein intensity estimates, protein iBAQ values, and protein statistics per group (CV, nr of missing)
prolfqua_dea	DE_.html	DE Analysis Report, with, e.g., interactive volcano plots
	QC_*.html	DE Quality Control Report
	ORA_*.txt	Files for all contrasts to perform ORA analysis
	GSEA_*.rnk	Files for all contrasts to perform GSEA analysis
	DE_*.xlsx	Spreadsheet file with protein abundance estimates, differential expression results, and additional summaries and statistics
	IBAQ_*.xlsx	Spreadsheet file with iBAQ protein abundances
	SummarizedExperiment.rds	Serialized SE object used by the *exploreDE* application

./prolfqua_qc.sh -i data_dir/ -d annotation.xlsx -s DIANN -o results_dir

If the QC shows outlier samples, e.g., samples with fewer peptides and proteins or samples that unexpectedly cluster with samples from a different group, or if there is a noticeable batch effect, we would edit the annotation file. For instance, we could remove rows with outlier samples or add an explanatory variable using the column *subject* to model the batch effect (see Table 3).

A configuration file with information about the experiment and analysis parameters is needed to run the differential expression analysis. Here, we specify, for instance, the normalization method, the protein intensity estimation method, or thresholds used to visualize significant fold changes. We create the configuration with default settings by calling the command: ./prolfqua_yaml.sh config.yaml

Finally, the *DEA* analysis can be run by calling the command:

./prolfqua_dea.sh -i data_dir/ -d annotation.xlsx -y config.yaml -s DIANN

In a workflow management setting, the annotation and YAML files are generated based on information stored in the LIMS system. For B-Fabric, we create the annotation and YAML files using the *bfabricPy* library.^48^

### Analyzing an Experiment with Two Factors

To demonstrate how we use *prolfquapp* to analyze a data set with two factors and repeated measurements, we used the renal cell carcinoma data set^46^ as an example. In this study treatment-naive tumors (T) and paired normal adjacent tissues (NAT) were collected in young and old patients. Table 1 shows the annotation file. It is worth noting that although this experiment has two factors, cell type, and age, we merge them, and the model uses a single explanatory variable, Group. In addition, we specify the patient identifier in the column Subject. We specified the model in this way because we were interested in looking into the interaction between age and cell type, and furthermore, we wanted to block for between patient differences.

Finally, we need to specify the contrasts, which we do by adding two more columns to the annotation file, one containing the name of the comparison (ContrastName) and the other a formula expressing the differences (Contrast). The first contrast T_vs_NAT examines the differences in tumor and adjacent tissue; the contrast Old_vs_Young, the difference between old and young; the Contrasts T_vs_Nat_gv_Young and T_vs_NAT_gv_Old the differences within the groups of young and old patients, while the last examines if this difference depends on the age of the patients (DoesTvsNatDependsOnAge). The full analysis is available to review the generated outputs from the “*DEA* Large Data set Example” web resource.^49^

### Outputs — Interactive HTML Report and Support of Downstream Analysis

The *prolfquapp* provides a comprehensive set of outputs, including interactive HTML reports, XLSX files, or serialized R objects, to facilitate data interpretation. Table 5 lists the output files generated by the QC and DEA applications.

**Figure 2:**
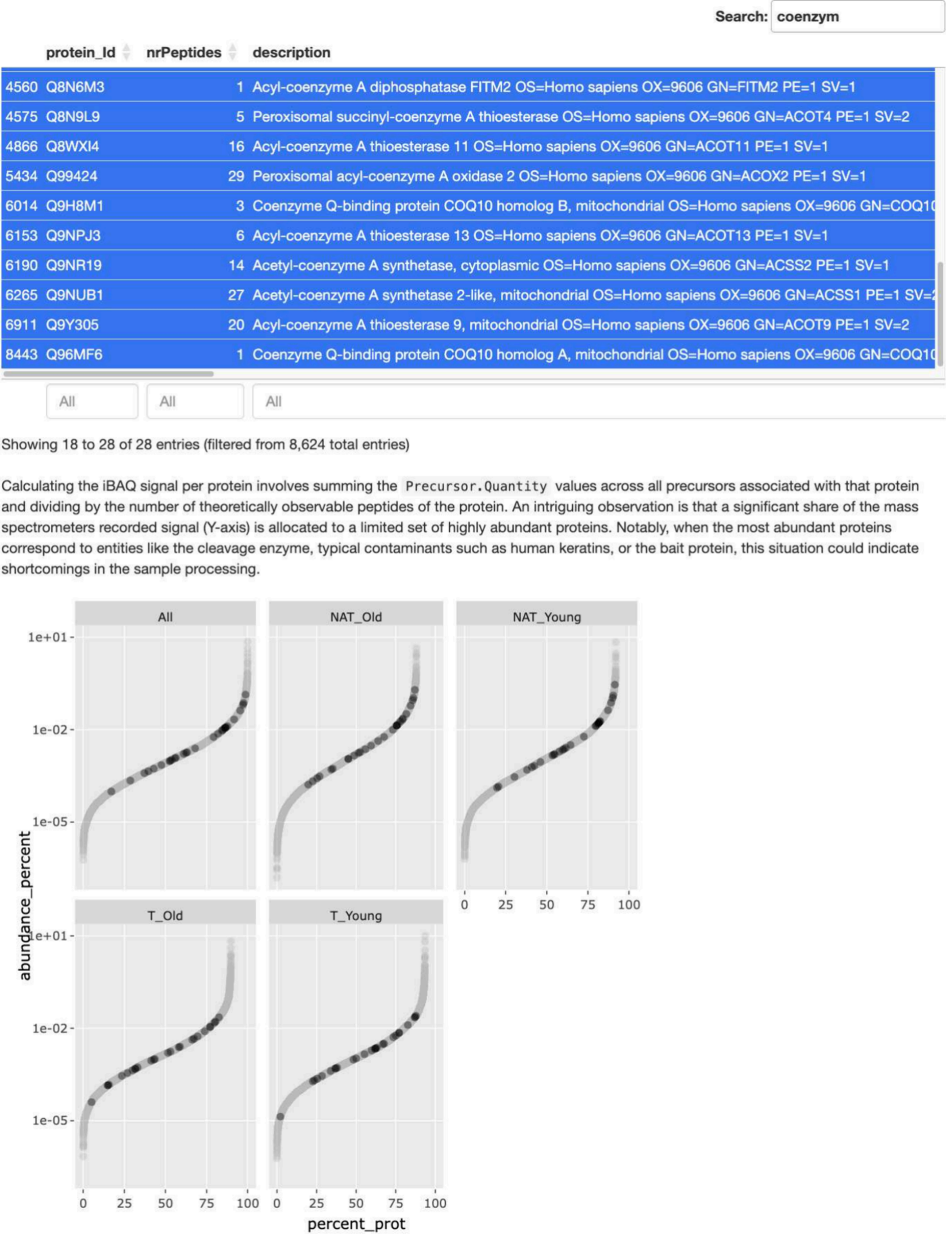
Screenshot of the interactive abundance visualization in the QC HTML report. The plot shows the protein iBAQ values for each group. The plot is interactive, allowing users to select proteins, for instance, all having the word coenzyme in the description line and highlighting them in the figure.

The QC application generates a QC and sample size estimation report and an interactive protein or peptide abundance report (Figure 2). Furthermore, we also write an XLSX file containing either peptide abundances, or protein abundance estimates determined using the Tukeys median polish, protein *iBAQ* values, and summaries such as the number of peptides per protein and experiment or sample. The protein quantity estimates are an alternative to the protein-level summaries produced by the quantification software, that typically reports *maxLFQ* abundances. Furthermore, using the values in the XLSX file, we can reproduce the figures in the QC report.

The *prolfquapp DEA* application generates a folder with two subfolders, one containing all the input files needed to replicate the analysis and a folder containing all the result files. The resulting folder can be archived, and the data in the input subfolder can be used to replicate or, by changing parameters or editing the annotation file, modify the analysis.

**Figure 3:**
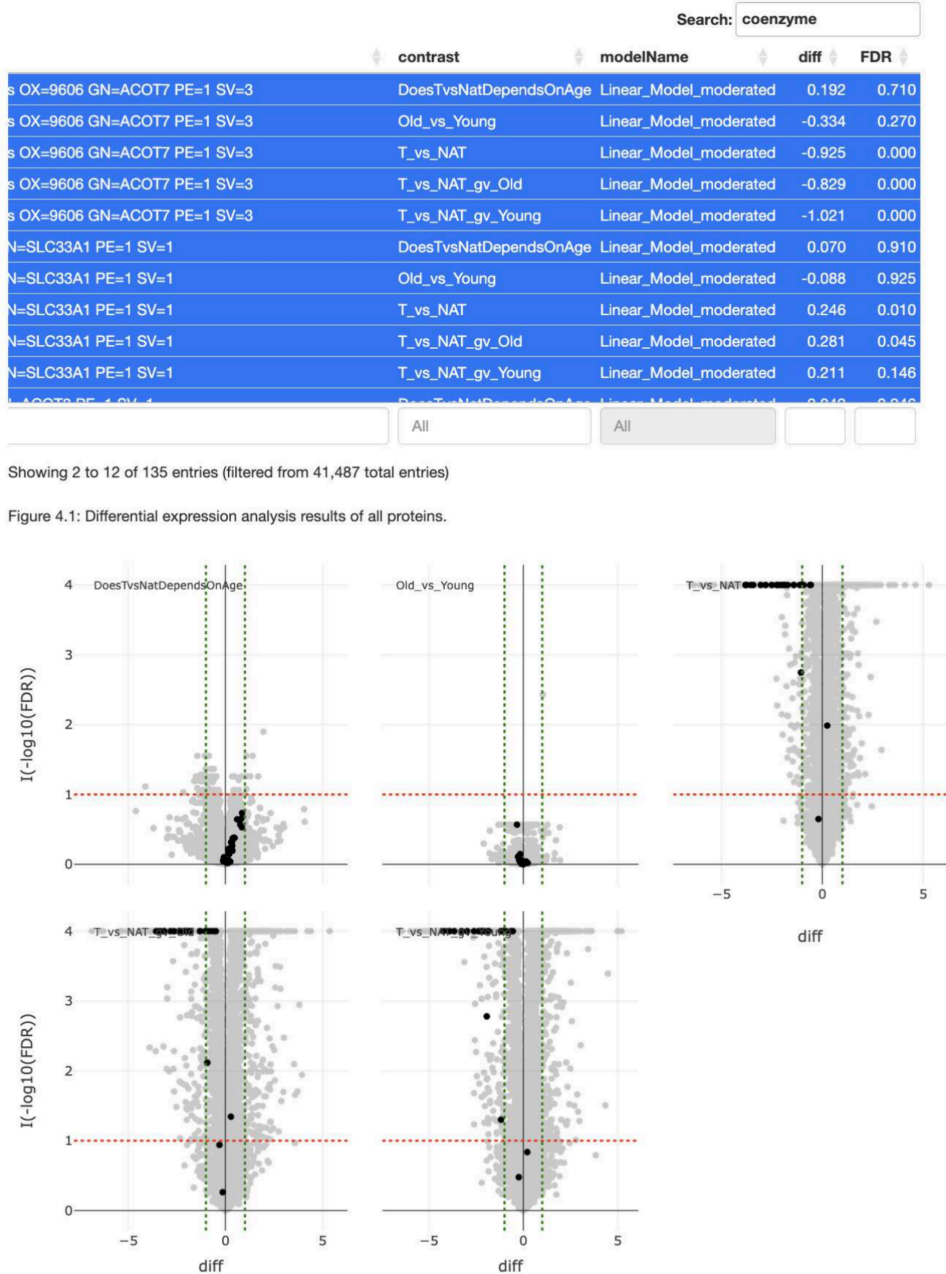
Interactive volcano plots in the DEA HTML report. The plot is interactive, allowing users to select proteins, for instance, all having the word coenzyme in the description line and highlighting them in the figure.

The primary output is the report in HTML format. The document starts by introducing differential expression analysis. Then, we show visual summaries of protein and peptide identification, such as the number of identified proteins or the distribution of missing observations. The report visualizes the results of the *DEA* using volcano plots for all studied contrasts. These volcano plots are interactive and linked when there are multiple comparisons. This functionality allows users to highlight a protein across all volcano plots by clicking on a data point, making it easier to identify quantitative and qualitative interactions, or search for proteins of interest in the table with the *DEA* results (see Figure 3). The report also includes dynamic tables that allow users to search and filter proteins of interest, which can be highlighted in the volcano plot. Next, we summarize the numbers of differentially expressed proteins and visualize them using an upset plot, which displays the intersections of significant proteins or peptides between multiple sets of contrasts (see Supporting Material 2). The section dedicated to additional analysis provides pathways for downstream omics analysis, such as gene set enrichment analysis, with direct links to web services like WebGestalt and the String functional protein association network.

In addition, we provided a *DEA*-QC HTML document with plots and tables, which we found informative but did not make it into the main report. The figures there, for instance, help to asses if the data normalization method was able to reduce the standard deviation of proteins within groups compared to only log2 transformed intensities (see Supporting Material 2). This document also contains histograms showing the distribution of the p-values for each contrast, an important plot helping to detect potential issues in a data set, such as biases or errors in the statistical model.

While the HTML report offers an interactive and visual exploration of the results, the main output of *prolfquapp* is an XLSX file. The XLSX file includes comprehensive tables with peptide and protein intensity estimates, IBAQ values, differential expression analysis results, and various protein or peptide summaries. These summaries provide essential metrics, such as the number of peptides per protein, peptide counts per sample, and protein coefficient of variation (CV), allowing for the filtering of protein coverage, proportion of missing values, or highly variable proteins. Spreadsheet programs remain a widely used tool among life science researchers and are ideal for filtering the results. Furthermore, figures and plots from the HTML report can be fully reproduced using the data contained in the XLSX output.

A conscious design choice was not to include, downstream analysis such as ORA or GSEA, or functionality to change colors in figures or figure layouts. The reason here is that there are excellent tools for ORA and GSEA such as *WebGestalt*,^50^*MSigDB*,^51^ or *string-db*(52) that can perform downstream analysis of omics data. Furthermore, we aim to run the application without user interactions and therefore we generate SE files compatible with *exploreDE*.

With its dual approach of offering both interactive HTML reports and data-rich XLSX files, *prolfquapp* is a versatile tool that can adapt to the needs of both exploratory and detailed analytical workflows.

### Interactive Visualization Using exploreDE

The importance of interactively visualizing the data, especially the ability to easily create publication-ready visualizations by life science experts, must be considered. Therefore, the seamless integration of *prolfquapp* with the *exploreDE* application is an essential cornerstone of the differential expression analysis pipeline. The *exploreDE* application allows the creation of highly customizable heatmaps, volcano, or PCA plots, which can be exported in various formats and included in presentations, reports, and publications. The Supporting Material 3(53) shows the interactive visualization of the renal cell carcinoma data set using the *exploreDE* application.

### Reproducibility and Replicability

*Prolfquapp* enhances reproducibility and replicability by generating self-contained outputs encapsulating all necessary parameters and settings. Lastly, it is essential to report all the steps performed, the execution progress, and errors for noninteractive applications. For this task, we use the R *logger* library.^54^

To simplify running *prolfquapp* and enhance reproducibility, we provide container images alongside the R package. If Docker or Podman is installed on your system, a script^55^ available in the repository will handle the setup and execution of the container, which allows users without experience in container technology to execute any of the command-line scripts within a versioned environment.

### Performance

**Figure 4:**
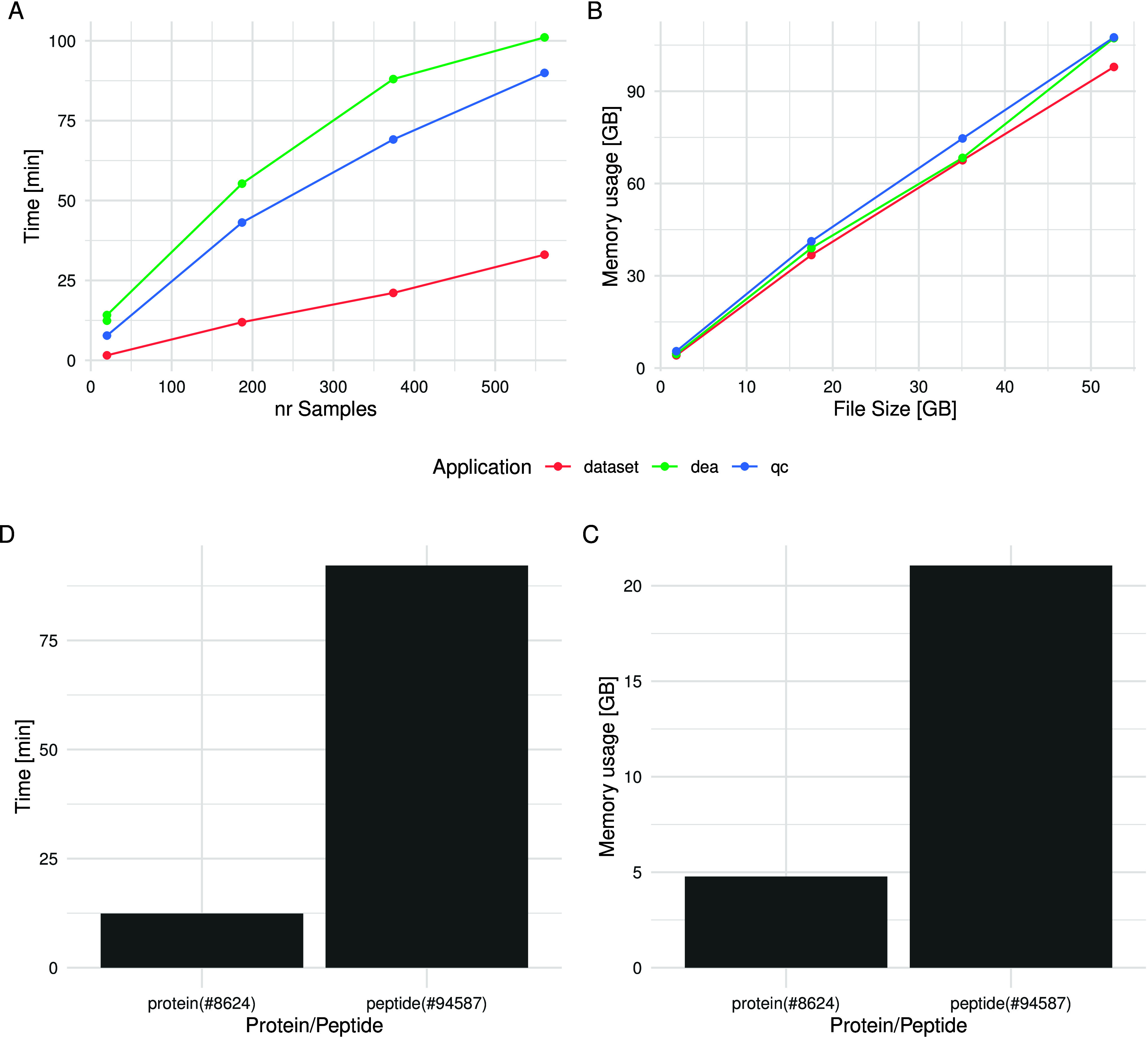
Panel A, Runtime of *prolfquapp* command-line applications (*prolfqua_data set*, *prolfqua_qc* and *prolfqua_dea*) as a function of the number of samples (20, 187 374, 561). Panel B, Memory usage of the *prolfquapp_dea* application as a function of the report.tsv file size. Panel C and D, Runtime and memory usage of the *prolfqua_dea*) application as a function of the number of proteins (8624) or peptides (94587), for a data set with 20 samples.

The renal cell carcinoma data set comprises 187 samples, with 8624 proteins and 94587 peptides, identified using the FragPipe DIA-NN workflow. To assess the performance of *prolfquapp* for different experiment sizes, we created three more data sets from this data set, one smaller with 20 samples, and two larger by duplicating (374 samples) and triplicating the original data set (561 samples). We then ran the *prolfqua_data set*, *prolfqua_qc* and *prolfqua_dea* applications on these data sets on a server with a *AMDEPYC*774264 – *CoreProcessor* and 1*TB* RAM. Since *R* runs as a single-threaded process, only one of the 64 cores is used. The runtime of the applications is shown in Figure 4. The application’s runtime increases proportional to the number of samples when the number of proteins is constant (see 4 Panel A). Also, the memory consumption of the *prolfquapp* applications is proportional to the file size (see 4 Panel B). The Barplot in Panel C in Figure 4 shows the runtime and Panel D the memory usage when performing protein level analysis, compared to peptide level analysis, for the data set with 20 samples. Here, the model fitting dominates the runtime. However, since the models are fitted independently for each peptide, there is potential for parallelization, which we plan to implement in future versions of the software. The results shown here were generated using the scripts provided as part of the Supporting Material 2 and can be replicated using Docker image of *prolfquapp* we provide (see Supporting Material 2).

### Software Availability

The *prolfquapp* package is available as open-source software and can be accessed via github repository.^56^ The package is compatible with Windows, macOS, and Linux operating systems. Instructions for installation, usage, and documentation are provided in the repository. The software is released under the MIT License, allowing free use, modification, and distribution. We encourage the community to report any issues or bugs via the GitHub issue tracker and welcome contributions in the form of code submissions or feature requests.

## Conclusion

Looking ahead, we are committed to expanding *prolfquapp*’s functionality to meet the evolving needs in proteomics data analysis. One planned feature is integrating the *prozor* R package^57^ to provide an additional option for determining peptide-to-protein relationships, offering users more flexibility in their analysis. We also plan to report peptide-level fold changes in addition to the protein-level fold changes by default, providing deeper insights and a more detailed understanding of differential expression results. Furthermore, we aim to offer more options to enhance the modeling of missing observations and include count-based models.

The *prolfquapp DEA* application creates organized folders containing all relevant inputs (annotation, parameters in YAML file) and outputs (e.g., results, reports), preserving all analysis components. Additionally, we provide Docker images to guarantee a consistent computational environment, allowing users to replicate the exact setup. In this way, *prolfquapp* helps scientists meet the requirements of funding agencies, journals, and academic institutions while publishing their data according to the FAIR data principles.

We designed *prolfquapp* so we can integrate it into scalable, high-throughput, and reproducible workflows by streamlining the configuration and execution. Its seamless integration with workflow management systems makes it particularly suitable for large-scale experiments where interactive analysis is impractical. By providing detailed, informative reports and interactive visualization of differential expression analysis results, *prolfquapp* lowers the barrier for end users to engage in advanced proteomics analysis. Furthermore, the integration with the *exploreDE* application allows the generation of publication-ready visualizations, giving the end users the independence to present their results. Through this, we made *prolfquapp* a practical solution for a wide audience within the proteomics community. Looking ahead, we anticipate that community contributions, such as bug reports and feature suggestions, will play an important role in shaping future versions of *prolfquapp*, helping ensure that it continues to meet the needs of the field as it evolves.

## Supporting Information Available

Supporting Material S1: Do FDR Estimates Differ When Filtering the Protein List Before or After Differential Expression Analysis?, Supporting Material S2: Example Analysis of a Data set with Two Factors Using prolfquapp, Supporting Material S3: *Prolfquapp DEA SummarizedExperiment* visualized with *exploreDE*(53) (PDF)

## Abbreviations Explanation

API: application programming interface
CV: Coefficient of Variation
DEA: Differential Expression Analysis
DIA: data independent acquisition
FAIR: Findable, Accessible, Interoperable, and Reusable
GSEA: Gene Set Enrichment Analysis
iBAQ: intensity-based Absolute Quantification
LC: Liquid Chromatography
LC-MS: Liquid Chromatography followed by Mass Spectrometry
MS: mass spectrometry
ORA: Over-Representation Analysis
PCA: Principal Component Analysis
QC: Quality Control
ROC: Receiver Operator Curve
SE: SummarizedExperiment
TMT: Tandem Mass Tag
YAML: YAML ain’t markup language
