# Supplementary material for: prolfquapp — A User-Friendly Command-Line Tool Simplifying Differential Expression Analysis in Quantitative Proteomics
Source: J Proteome Res. 2025 Jan 24;24(2):955–65. doi: 10.1021/acs.jproteome.4c00911 (PMC11812002; doi:10.1021/acs.jproteome.4c00911)
Supplement: Supplementary file 1 — pr4c00911_si_001.pdf [file pr4c00911_si_001.pdf]

Supporting Information: prolfquapp - A User-Friendly Command-Line Tool Simplifying Differential Expression Analysis in Quantitative Proteomics

Witold E. Wolski[1,2,*] Jonas Grossmann[1,2] Leonardo Schwarz[1,2] Peter Leary[1,2]
Can Turker[1] Paolo Nanni[1] Ralph Schlapbach[1] Christian Panse[1,2]

[1] Functional Genomics Center Zurich (FGCZ), ETH Zurich / University of Zurich, Winterthurerstrasse 190, 8057 Zurich, Switzerland
[2] Swiss Institute of Bioinformatics (SIB), Quartier Sorge - Batiment Amphipole, 1015 Lausanne, Switzerland

[*] Correspondence: Witold E. Wolski <witold.wolski@fgcz.uzh.ch>

Contents

***Supporting Material S1*: Do FDR Estimates Differ When Filtering the Protein List Before or After Differential Expression Analysis?**

***Supporting Material S2*: Example Analysis of a Dataset with Two Factors Using prolfquapp**

***Supporting Material S3*: Prolfquapp DEA SummarizedExperiment visualized with exploreDE**

Supporting Material S1: Do FDR Estimates Differ When Filtering the Protein List Before or After Differential Expression Analysis?

Contents

Introduction . . . 1

The computer Experiment **1**

Example 1, filtering without changing the distribution of p-values . . . 1

Example 2, filtering and changing the distribution of p-values. . . . 4

Measured data Example **6**

Example 1, No missing modeling . . . 6

Example 2, With missing modeling . . . 6

Introduction

Some may assume that shortening the list of proteins would artificially increase the false discovery rate (FDR). However, the FDR, estimated using the Benjamini-Hochberg correction, depends primarily on the p-value distribution relative to the distribution of p-values under the null hypothesis (H_0, a uniform distribution), not the absolute number of proteins tested. While methods controlling the Family-Wise Error Rate (FWER) are sensitive to the total number of tests, the FDR estimation is unaffected as long as the p-value distribution remains unchanged.

The computer Experiment

Example 1, filtering without changing the distribution of p-values

We start by simulating a list of $m = 3000$ p-values. 90% datapoints comes from H0, 10% from H1 (fold change of 2). The group sizes are 4. We store these p-values in the array $pvals24$.

```
m <- 3000
simulate.p.values <- function(
    i, delta = 2, fraction = 0.1, ss = 4){
  control <- rnorm(ss,0,1)
  treatment <- rnorm(ss,0,1)
  if (runif(1) < fraction)
    treatment <- treatment + delta
  return(t.test(treatment,control)$p.value)
}
pvals24 <- sapply(1:m, simulate.p.values,
                  delta = 2, fraction = 0.4 )
```

We plot the distribution of the p-values and see that we can estimate FP, TP, FN and TN from the histogram, which allows us to compute the false discovery proportion (FDP).

$$FDP = \frac{FP}{FP + TP}$$

```r
hist(pvals24, breaks = 20, ylim = c(0,m/3), main = "B")
abline(h = (m - m * 0.4)/20, col = 3)
abline(v = 0.05, col = 2)
text(x = c(0.02, 0.02, 0.2, 0.2),y = c(20,100,20,100), labels = c("FP","TP","TN","FN"))
```

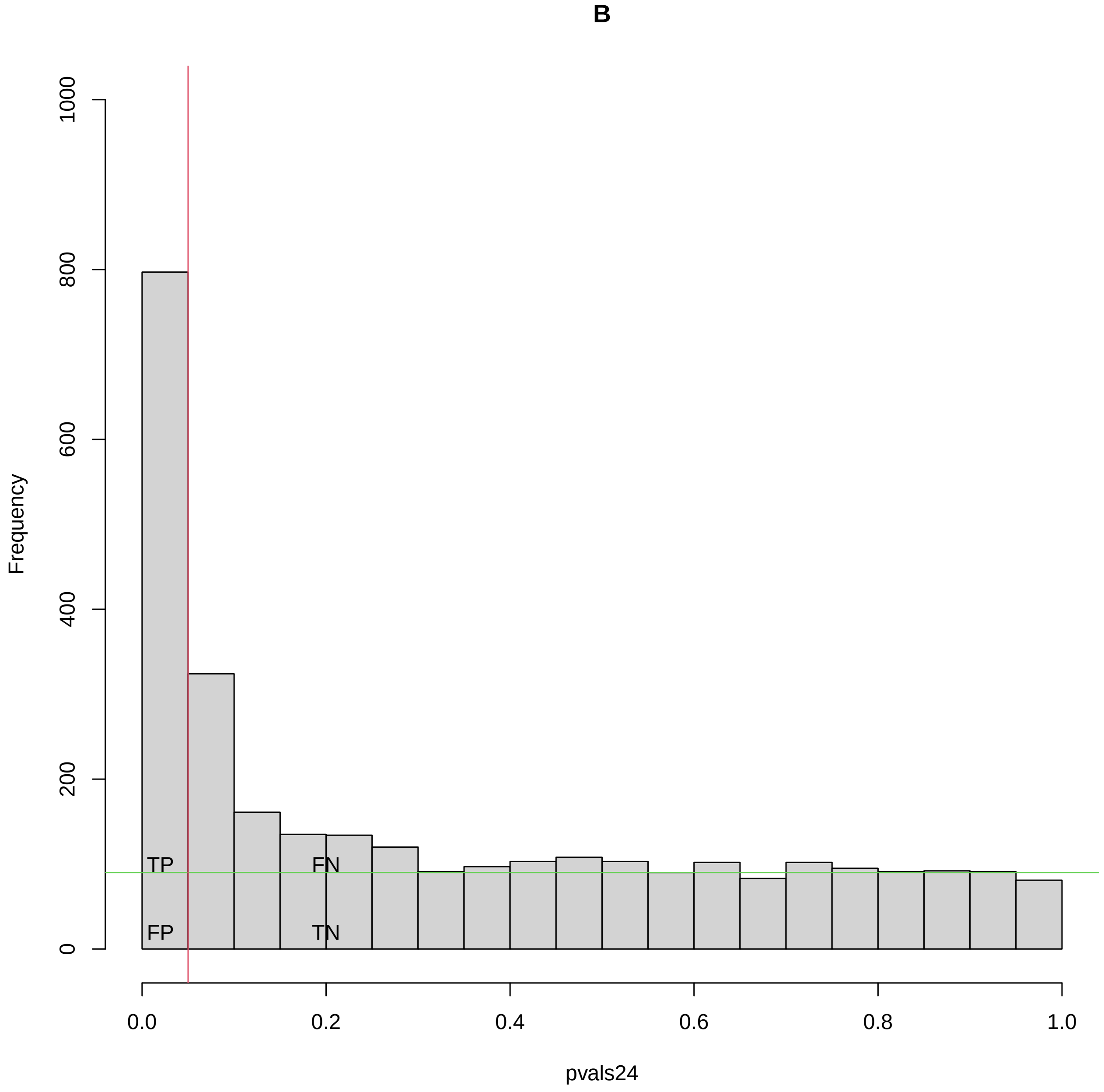

Distribution of p-values. Red line indicates the size of test at 5%, while the green line the uniform distribution of the p-values when $H0$ true.

We can also compute the FDR using the Benjamini-Hochberg method using the function *p.adjust*.

```r
xx <- data.frame(p.vals = pvals24, FDR = p.adjust(pvals24, method = "BH"))
```

In proteomics experiments, up to 1/3 of proteins are identified by a single peptide. If we remove these proteins from the list and keep only those quantified by two or more peptides, do we need to recompute the FDR? We filter the simulated p-values and keep only 2/3 of them. We randomly choose which data we hold. We then

compute the FDR from the remaining $p - values$.

```
xx <- xx[sample(1:m,floor(m*2/3)),]
xx$FDR2 <- p.adjust(xx$p.vals, method = "BH")
```

We compare the FDR estimates computed for all 3000 proteins and the 2000 proteins left after filtering. We can see that the differences in the p-value estimates are less than 1.

```
par(mfrow = c(1,2))
xx <- xx[order(xx$p.vals),]
with(xx,plot(FDR,FDR2, xlab = "FDR", ylab = "FDR2"))
abline(c(0,1), col = 2)
with(xx,plot(FDR, (FDR - FDR2)*100, pch = ".", type = "l"))
abline(h = 0)
```

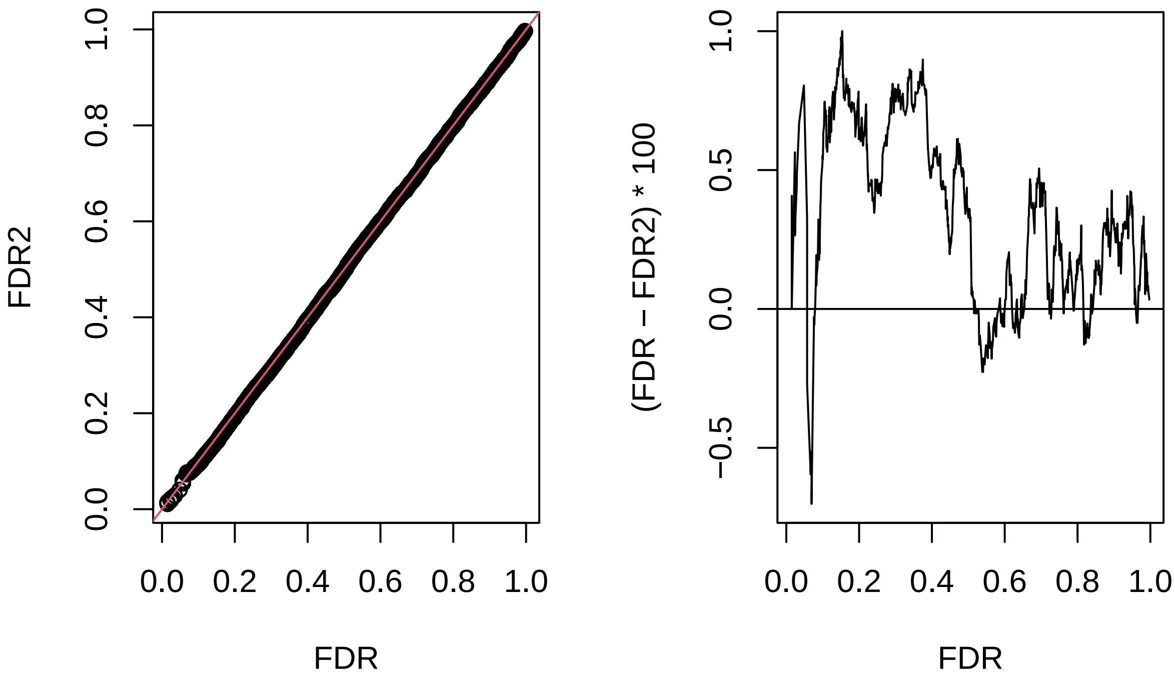

Left Panel: FDR estimates computed for a random subset (FDR2), as a function of the FDR estimates computed for the entire list. Right Panel: Difference between FDR2 and FDR estimates.

We examine how this affects the list of selected proteins if we filter for FDR at 5%, 10%, or 25%.

```
ll <- data.frame(FDR = c("5" = sum(xx$FDR < 0.05),
                         "10" = sum(xx$FDR < 0.1),
                         "25" = sum(xx$FDR < 0.25)),
                 FDR2 = c("5" = sum(xx$FDR2 < 0.05),
                          "10" = sum(xx$FDR2 < 0.1),
                          "25" = sum(xx$FDR2 < 0.25)))
knitr::kable(ll, caption = "Number of proteins/peptides selected when filtering for FDR < 5, 10 or 25 %
```

Number of proteins/peptides selected when filtering for FDR < 5, 10 or 25 %.

	FDR	FDR2
5	16	16
10	235	265
25	723	735

	FDR	FDR2

We see that we end up with almost identical lists of proteins, and it does not matter if we used the FDR computed before or after removing 1/3 of observations.

Example 2, filtering and changing the distribution of p-values.

We augment the original list of 3000 p-values with 10 p.values equal to 1 and compute the FDR. We change the distribution of the p-values. We remove those p-values equal 1 and 33% of the other p-values, leaving 2000 p-values. Finally, we recompute the FDR from the p-values in the filtered list.

```
addP <- ceiling(m*0.1)
pvals240 <- c(pvals24, rep(1,addP))
xx0 <- data.frame(p.value = pvals240, FDR = p.adjust(pvals240, method = "BH"))
```

```
xx0F <- xx0[-(3001:(3000 + addP)),]
xx0F <- xx0F[sample(1:m,floor(m*2/3)),]
```

Clearly, the p-value distributions do differ before and after filtering.

```
par(mfrow = c(1,2))
with(xx0,hist(p.value, main = "Starting list"))
with(xx0F, hist(p.value, main = "Filtered list"))
```

Now we recompute the FDR from the truncated list of p-values and compare the FDR estimates computed before with those computed after filtering the lists.

```
xx0F$FDR2 <- p.adjust(xx0F$p.value, method = "BH")
par(mfrow = c(1,2))
with(xx0F, plot(FDR, FDR2, xlab = "FDR", ylab = "FDR2"))
abline(0,1,col = 2)
with(xx0F, plot(FDR2,(FDR2 - FDR)*100, xlim = c(0,1), xlab = "FDR", ylab = "FDR2 - FDR"))
```

The difference between the FDR computed before and after filtering is larger. Again, we examine, how this affects the list of selected proteins if we filter for FDR at 5%, 10% or 25%.

```
ll <- data.frame(FDR = c("5" = sum(xx0F$FDR < 0.05),
                         "10" = sum(xx0F$FDR < 0.1),
                         "25" = sum(xx0F$FDR < 0.25)),

                 FDR2 = c("5" = sum(xx0F$FDR2 < 0.05),
                          "10" = sum(xx0F$FDR2 < 0.1),
                          "25" = sum(xx0F$FDR2 < 0.25)))
knitr::kable(ll, caption = "Number of proteins/peptides selected when filtering for FDR < 5, 10 or 25 %
```

Number of proteins/peptides selected when filtering for FDR $<$ 5, 10 or 25 %.

	FDR	FDR2
5	11	13
10	137	223
25	671	713

The lists of proteins/peptides filtered by FDR at 5%, 10%, and 25 have different lengths. These differences become relatively smaller when FDR thresholds are larger.

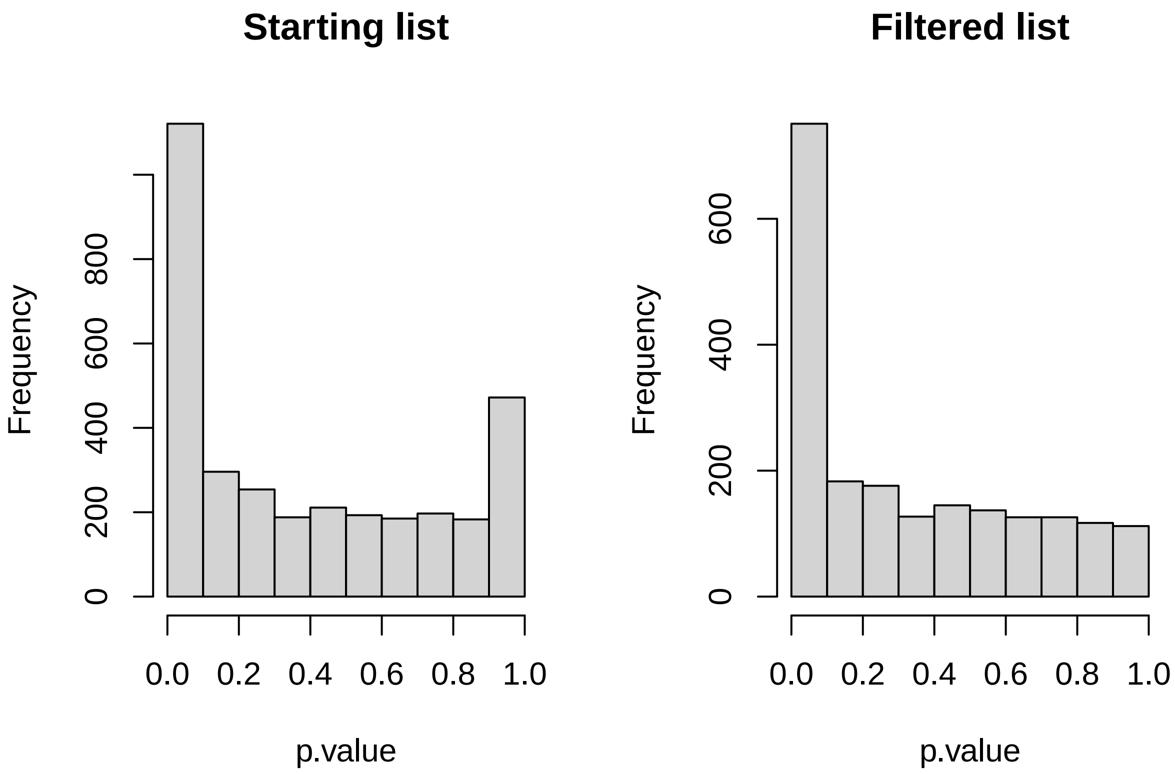

Distribution of p-values before and after filtering. Left panel: p-values before filtering. Right panel: p-values after filtering.

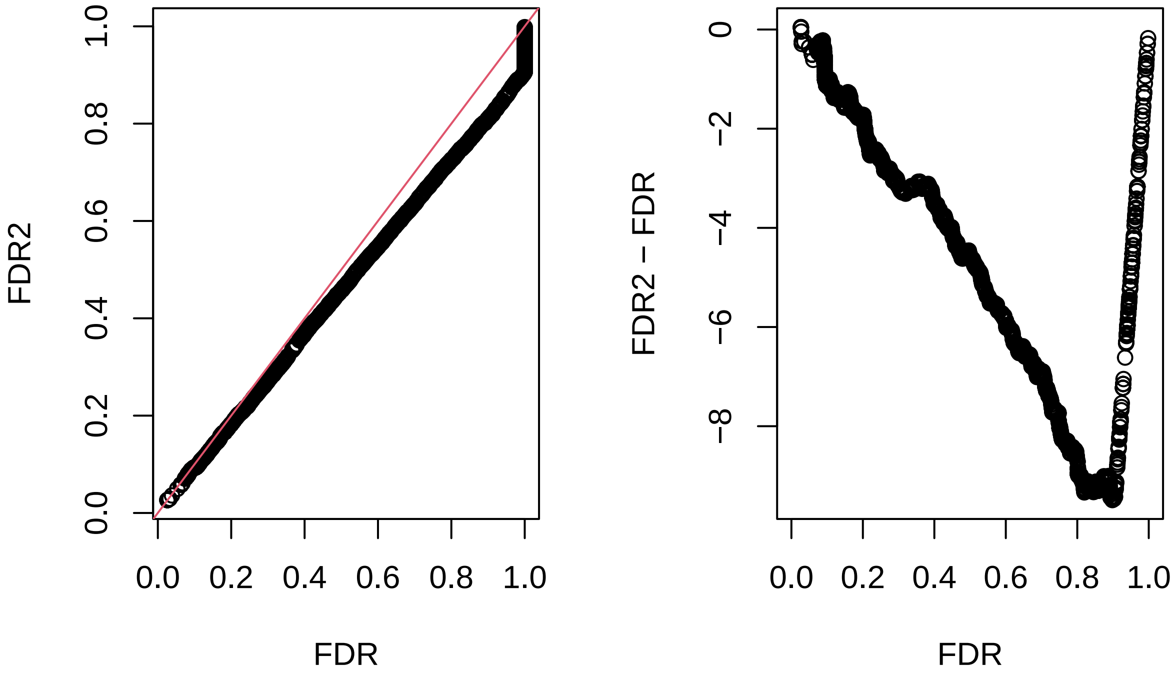

Left Panel: FDR estimates computed for the filtered subset (FDR2), as a function of the FDR estimates computed for the entire list. Right Panel: Difference between FDR2 and FDR estimates.

Measured data Example

For these two examples, we use the same data as in the main text. We have run differential expression analysis on a dataset without and with missing modeling. Missing data modeling and peptide filtering can be enabled in the *prolfquapp* configuration file. For details on how the data was generated, see the Supporting information S2.

Example 1, No missing modeling

We did run *prolfqua_dea* either on all proteins or when filtering for two and more peptides, and disabled missigness modelling.

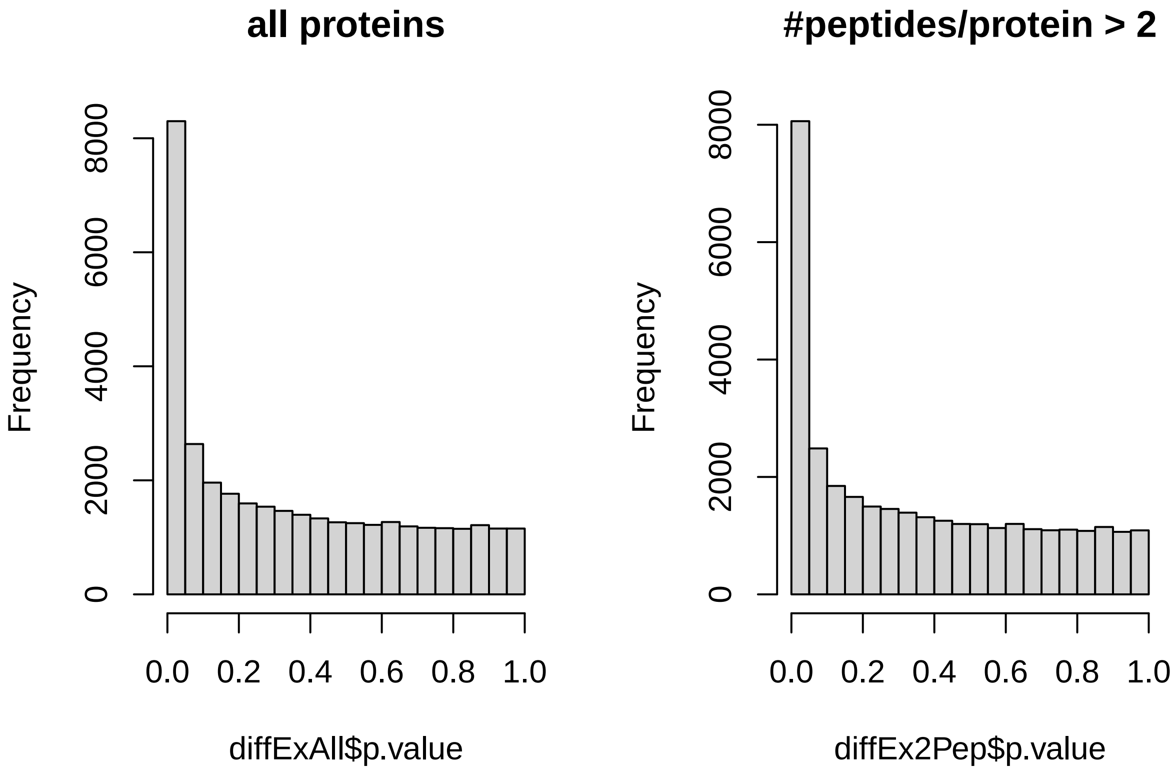

Histogram of p-values. Left Panel: p-value distrubtion for all proteins. Right Panel: p-value distribution for proteins with two or more peptides.

We compare the FDR estimates computed for all proteins, and for proteins identified by two or more peptides, using a scatterplot and see that there are very similar.

We obtain an almost identical list of proteins when filtering for an FDR threshold, independent of whether we filtered for two or more peptides before or after running the differential expression analysis.

Example 2, With missing modeling

We did run *prolfqua_dea* application, with missing data modeling, either on all proteins or when filtering for two or more peptides. We compare the FDR estimates obtained using either option.

We compare the FDR of proteins in both datasets using a scatterplot and see that the FDR are similar.

We obtain an almost identical list of proteins when filtering for an FDR threshold, independent of whether we filtered for two or more peptides before or after running the differential expression analysis.

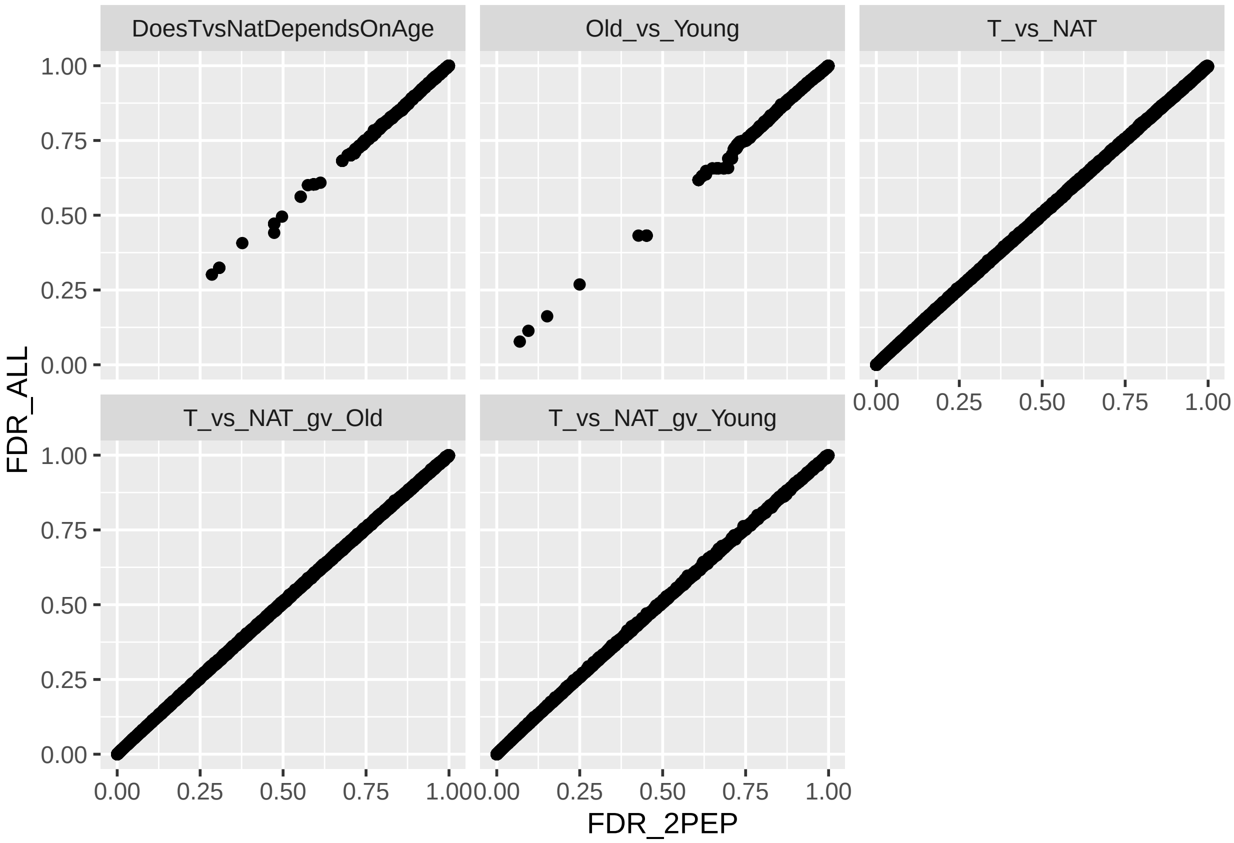

Scatterplot of FDR estimates computed for all proteins and for proteins identified by two or more peptides.

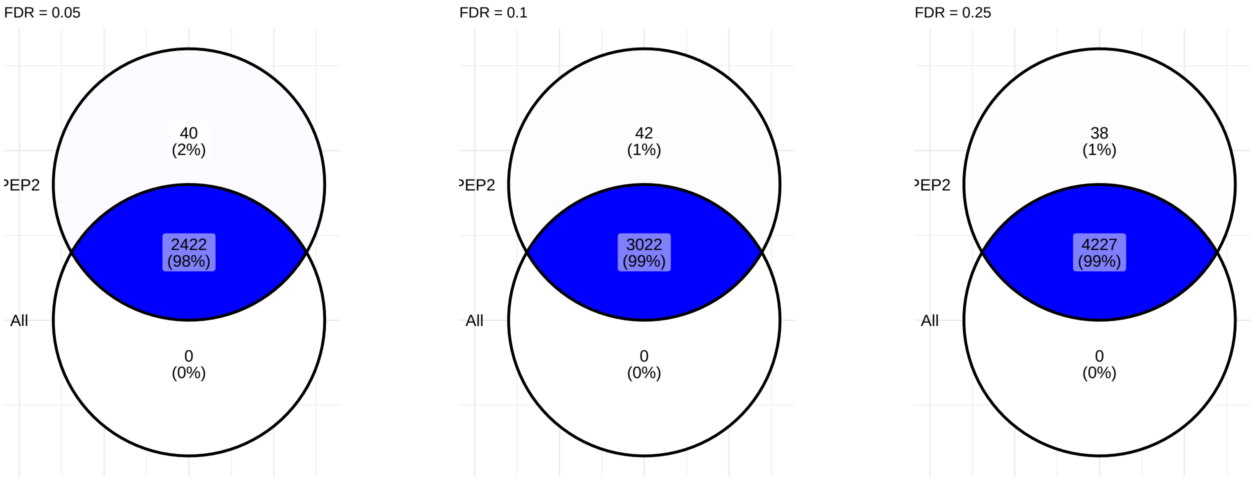

Venn diagrams comparing the lists of proteins we obtain when filtering the FDR estimates computed before and after filtering using the 5%, 10% and 25% FDR threshold

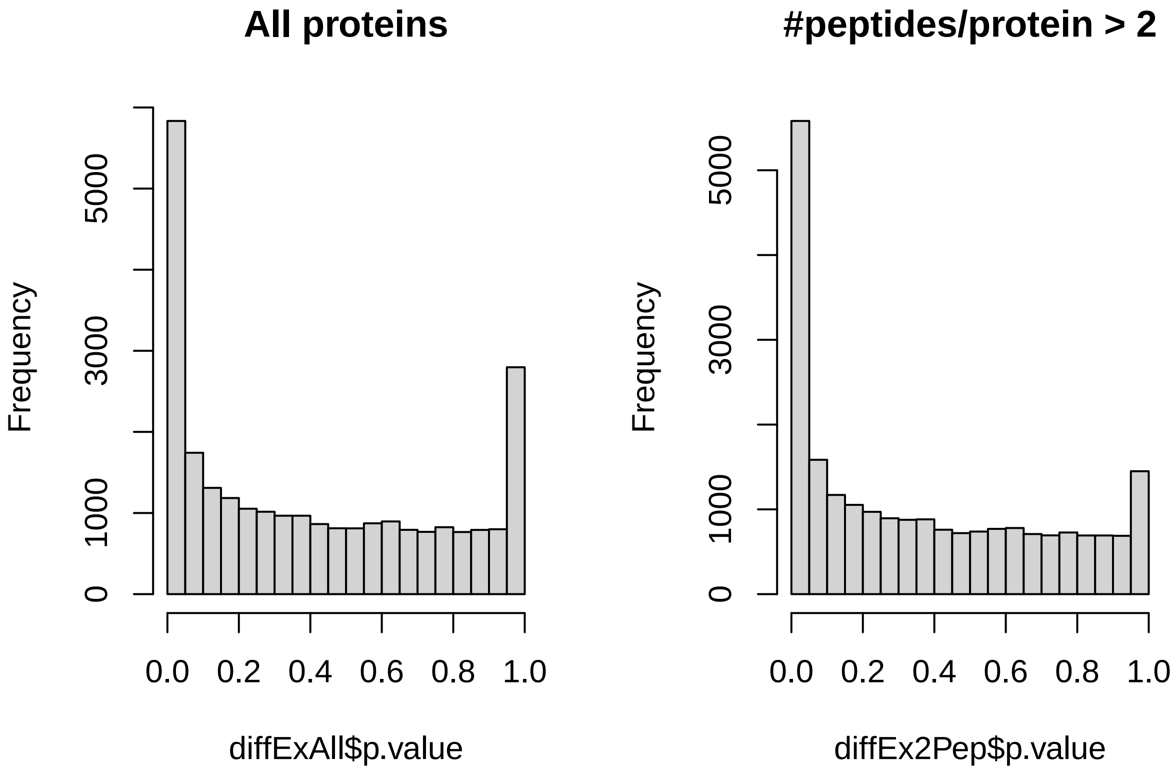

Histogram of p-values. Left Panel: p-value distribution for all proteins. Right Panel: p-value distribution for proteins with two or more peptides.

We obtain an almost identical list of proteins when filtering for an FDR threshold, independent of whether we filtered for two or more peptides before or after running the differential expression analysis.

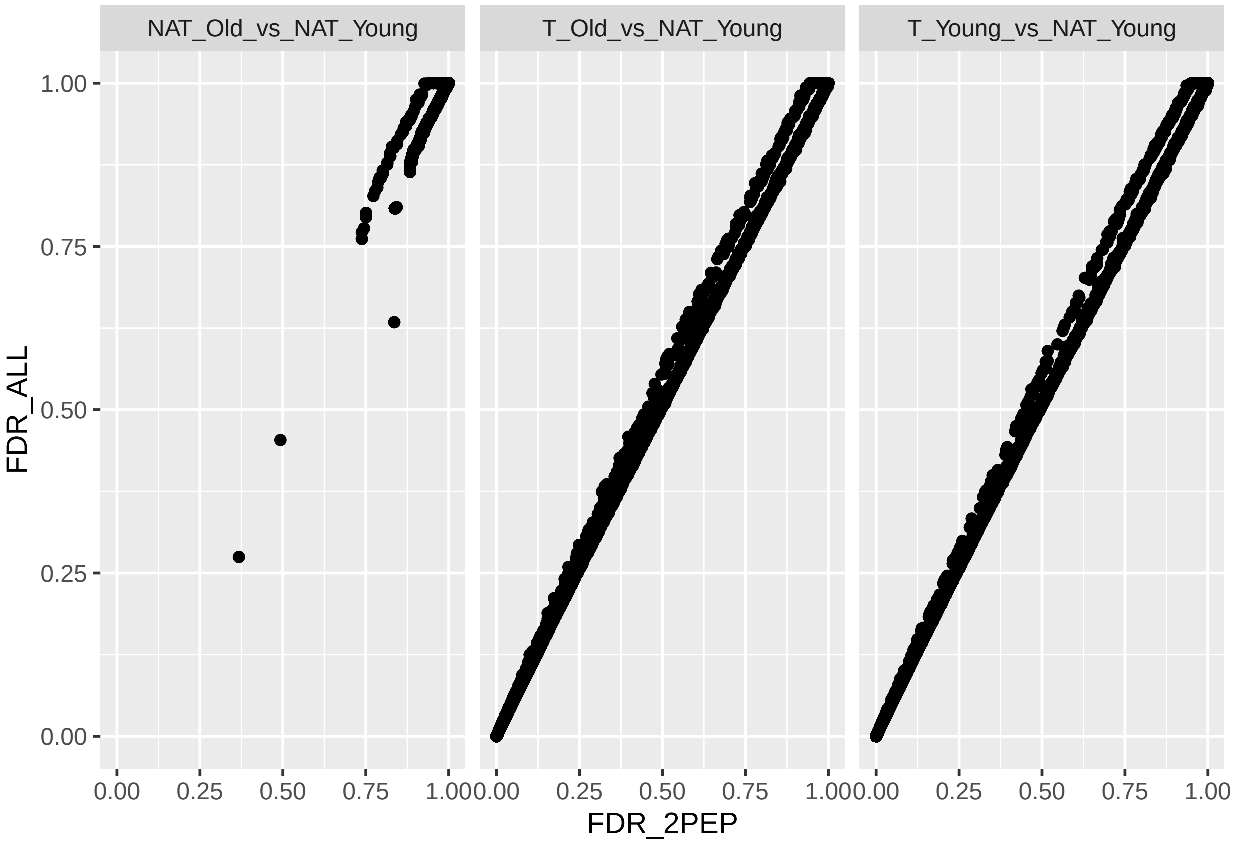

Scatterplot of FDR estimates computed for all proteins and proteins identified by two or more peptides.

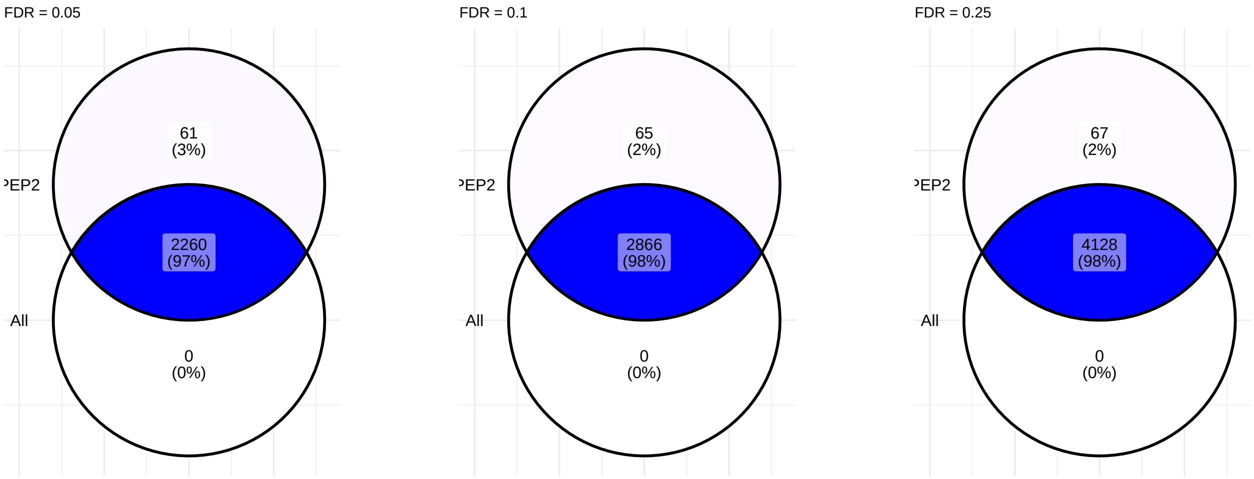

Venn diagrams comparing the lists of proteins we obtain when filtering the FDR estimates computed before and after filtering using the 5%, 10%, and 25% FDR threshold.

Supporting Material S2: Example Analysis of a Dataset with Two Factors Using prolfquapp

Example analysis of RMSV000000696.1 dataset using prolfquapp

prolfquapp [https://github.com/prolfqua/prolfquapp] is an R package which can be used to perform differential expression analysis of proteins. It builds upon the core functionalities of the *prolfqua* package [https://github.com/fgcz/prolfqua].

The data used in this analysis we sourced from MassIVE Reanalysis - RMSV000000696.1. Two key files `report.tsv` and `msstats.tsv` were processed for this study, each approximately 17GB in size. These files were produced from the raw files using the FragPipe DIA workflow https://fragpipe.nesvilab.org/docs/tutorial_DIA.html, and the preprocessing is discussed in more detail in the publication by D.Kohler et al. https://doi.org/10.1038/s41596-024-01000-3.

The dataset has 187 samples and for more information about the dataset, we refer you to https://pdc.cancer.gov/pdc/study/PDC000200. The FragPipe DIA workflow identified and quantified 8613 Proteins.

We have chosen this dataset is to demonstrate the performance and functionality of the *prolfquapp* package to analyse large datasets and experiments with factorial designs.

Replicating the document

The web-resource https://fgcz-proteomics.uzh.ch/public/wew_prolfquapp/DEA_large_example/ contains copies of the input `report.tsv` file and `msstats.tsv` file, and examples of `.yml` files and annotation XLSX files which the *prolfquapp* application requires as input. It also contains the outputs generated by *prolfquapp*.

To recreate the analysis on your computer you will need the docker application. Furthermore, you need to download the content of the folder. Then run:

By executing:

```
./prolfquapp_docker.sh quarto render Readme.qmd --to html
```

you execute all the code blocks in this document. The shell script `prolfquapp_docker.sh` file is used to set up the docker container and run the analysis using the *prolfquapp* installation provided by the docker image.

The folders starting with `FragPipe_` contain the quantification results. Folders starting with `qc_` contain the output of the `prolfqua_qc` application while folders starting with `DEA_` contain the output of the `prolfqua_dea` application.

The latest version of the document is available at: https://github.com/wolski/DEA_large_dataset_example

Creating a Subset of 20 files

Using the R code below we created a subset containing data only from 20 samples out of the 187 samples.

```
library(readr)
alld <- readr::read_csv("FragPipe_f187_msstats/msstats.csv")
Runs <- unique(alld$Run)
Runs20 <- sample(Runs, 20)
ms20 <- alld[alld$Run %in% Runs20,]
readr::write_csv(ms20, file = "FragPipe_f20_msstats/msstats20.csv")
```

```
alldiann <- readr::read_tsv("FragPipe_f187_diann/report.tsv")
Runs <- alldiann$File.Name |> unique()
Runs20 <- sample(Runs , 20)

ms20diann <- alldiann[alldiann$File.Name %in% Runs20,]
readr::write_tsv(ms20diann, file = "FragPipe_f20_diann/f20_report.tsv")
```

Duplicating and triplicating the dataset

To asses the runtime and memmory usage for larger experiments we duplicte and triplicate the original dataset.

```r
alld <- readr::read_tsv("FragPipe_f187_diann/report.tsv")
alld2 <- alld
alld2$Run <- paste0(alld2$Run, "_V2")
dd <- unique(alld2$File.Name)
alld2$File.Name <- gsub("\\.mzML", "_V2\\.mzML", alld2$File.Name)
alldx <- dplyr::bind_rows(alld, alld2)
readr::write_tsv(alldx, "FragPipe_f374_diann/x2_report.tsv")
```

```r
alld3 <- alld
alld3$Run <- paste0(alld3$Run, "_V3")
alld3$File.Name <- gsub("\\.mzML", "_V3\\.mzML", alld3$File.Name)
alld <- dplyr::bind_rows(alldx, alld3)
dir.create("FragPipe_f561_diann")
readr::write_tsv(alld, "FragPipe_f561_diann/x3_report.tsv")
```

We here define a helper function to compute the run-time and memory usage of the QC and DEA analysis.

```r
#! label: computeruntime.

runtime_DEA <- function(log_file) {
  datafLOG <- read.table(log_file, header = TRUE, sep = "", fill = TRUE,
                          comment.char = "", check.names = FALSE,
                          skip = 0)
  datafLOG <- datafLOG[!grepl("^%CPU", datafLOG$`%CPU`), ]
  datafLOG <- datafLOG[-nrow(datafLOG),]
  datafLOG$TIME  <- as.numeric(lubridate::hms(datafLOG$TIME))
  datafLOG$GB <- as.numeric(datafLOG$RSS)/(1024*1024)
  res <- list(data = datafLOG, maxGB = max(datafLOG$GB),
              maxTime = max(datafLOG$TIME) / 60)
  return(res)
}
```

Setup

We start by cleaning the outputs of previous runs.

```bash
rm -f *.Rmd
rm -f prolfqua_*.sh
rm -rf DEA_*
```

```
rm -rf qc_*
rm -f FragPipe_f187_msstats/*.log
rm -f FragPipe_f20_msstats/*.log
rm -f FragPipe_f187_diann/*.log
rm -f FragPipe_f20_diann/*.log
rm -f FragPipe_f374_diann/*.log
rm -f FragPipe_f561_diann/*.log
```

Next we deploy the shell scripts provided by the *prolfquapp* package.

```
R --vanilla -e "prolfquapp::copy_shell_script(workdir = '.')"
```

DIANN reports.tsv file for 20 samples

Create dataset annotation file using information in the `mstats.tsv` file.

```
./prolfqua_dataset.sh -s DIANN -i FragPipe_f20_diann \
  -d FragPipe_f20_diann/dataset_diann_example.xlsx
```

Create QC and sample size estimation report, and generate XLSX file with protein abundance estimates using Tukeys median polish and iBAQ values. The outputs are stored in the `qc_dir_f20_diann` folder.

```
./prolfqua_qc.sh -s DIANN -i FragPipe_f20_diann \
  -d dataset_all_parallel.xlsx -o qc_dir_f20_diann
```

Example of DEA with parallel group design, with missingness modeling

```
./prolfqua_dea.sh -s DIANN -i FragPipe_f20_diann -d dataset_all_parallel.xlsx \
  -y config_model_missing_vsn.yml -w f20_diann_with_subject
```

Same analysis as before, but we filter for at least 2 peptides per protein.

```
./prolfqua_dea.sh -s DIANN -i FragPipe_f20_diann -d dataset_all_parallel.xlsx \
  -y config_2_or_more_missing.yml -w f20_diann_with_subject_2PEP
```

Example of DEA with factorial design, and no missingness modeling

```
./prolfqua_dea.sh -s DIANN -i FragPipe_f20_diann \
  -d dataset_all_interaction_no_Subject.xlsx \
  -y config_vsn.yml -w f20_diann_with_interaction
```

We rerun the same analysis but filter for two peptides per protein. The filter parameters are defined in the yaml file

```
ext_reader:
  extra_args: list(q_value = 0.01, hierarchy_depth = 1, nr_peptides = 2)
  preprocess: prolfquapp::preprocess_DIANN
  get_files: prolfquapp::get_DIANN_files
```

```
./prolfqua_dea.sh -s DIANN -i FragPipe_f20_diann \
  -d dataset_all_interaction_no_Subject.xlsx \
  -y config_2_or_more.yml -w f20_diann_with_interaction_2Peptides
```

Example PEPTIDE level analysis

Note that we use the `DIANN_PEPTIDE` option here.

```
./prolfqua_dea.sh -s DIANN_PEPTIDE -i FragPipe_f20_diann \
  -d dataset_all_parallel.xlsx -y config_model_missing_vsn.yml \
  -w f20_diann_peptide_with_subject
```

```
undebug(runtime_DEA)
resDS <- runtime_DEA("FragPipe_f20_diann/prolfqua_logMemUsage_dataset.log")
resQ <- runtime_DEA("FragPipe_f20_diann/prolfqua_logMemUsage_qc.log")
resD1 <- runtime_DEA("FragPipe_f20_diann/prolfqua_logMemUsage_dea_2.log")
resD2 <- runtime_DEA("FragPipe_f20_diann/prolfqua_logMemUsage_dea_1.log")
resDPEP <- runtime_DEA("FragPipe_f20_diann/prolfqua_logMemUsage_dea.log")

timing <- data.frame(name = c("Dataset", "QC", "DEA1", "DEA2","DEAPEP"),
  RAM_GB = c(resDS$maxGB, resQ$maxGB, resD1$maxGB , resD2$maxGB, resDPEP$maxGB),
  Time_min = c(resDS$maxTime, resQ$maxTime, resD1$maxTime , resD2$maxTime, resDPEP$maxTime))

knitr::kable(timing,caption = "f20 DIANN report tsv input")
```

f20 DIANN report tsv input

name	RAM_GB	Time_min
Dataset	4.060852	1.600000
QC	5.466263	7.666667
DEA1	4.606457	8.816667
DEA2	4.625717	9.583333
DEAPEP	22.207657	69.816667

DIANN reports.tsv file for 187 samples

- generating a dataset

```
./prolfqua_dataset.sh -s DIANN -i FragPipe_f187_diann \
  -d FragPipe_f187_diann/dataset_diann_example.xlsx
```

- generating a qc report

```
./prolfqua_qc.sh -s DIANN -i FragPipe_f187_diann \
  -d dataset_all_parallel.xlsx -o qc_dir_f187_diann
```

- generating a DEA with factorial design

```
./prolfqua_dea.sh -s DIANN -i FragPipe_f187_diann \
  -d dataset_all_interaction_no_Subject.xlsx \
  -y config_vsn.yml -w f187_diann_with_interaction
```

```
resDS <- runtime_DEA("FragPipe_f187_diann/prolfqua_logMemUsage_dataset.log")
resQ <- runtime_DEA("FragPipe_f187_diann/prolfqua_logMemUsage_qc.log")
resD2 <- runtime_DEA("FragPipe_f187_diann/prolfqua_logMemUsage_dea.log")

timing <- data.frame(name = c("Dataset", "QC", "DEA1"),
  RAM_GB = c(resDS$maxGB, resQ$maxGB,resD2$maxGB),
  Time_min = c(resDS$maxTime, resQ$maxTime, resD2$maxTime))

knitr::kable(timing,caption = "f187 DIANN report tsv input")
```

f187 DIANN report tsv input

name	RAM_GB	Time_min
Dataset	36.36521	10.95000
QC	41.42694	42.58333
DEA1	41.48651	47.80000

MSstats.tsv formatted output for dataset with 20 files.

$FragPipe$ can reformat the $DIA - NN$ output in report.tsv to $MSstats$ compatible output. We show here that you can run a the same steps as above output using $prolfquapp$.

Create dataset annotation file based on information in the `mstats.tsv` file.

```
./prolfqua_dataset.sh -s MSSTATS -i FragPipe_f20_msstats \
  -d FragPipe_f20_msstats/dataset_msstats20_example.xlsx
./prolfqua_qc.sh -s MSSTATS_FP_DIA -i FragPipe_f20_msstats \
  -d dataset_all_parallel.xlsx -o qc_dir_msstats20
```

- generate DEA with factorial design

```
./prolfqua_dea.sh -s MSSTATS_FP_DIA -i FragPipe_f20_msstats \
  -d dataset_all_interaction_no_Subject.xlsx \
  -y config_vsn.yml -w f20_msstats_with_interaction_no_subject
```

DIANN report.tsv with 374 samples.

This dataset was create by duplicating the dataset with 187 samples.

- generate dataset file to annotate samples

```
./prolfqua_dataset.sh -s DIANN -i FragPipe_f374_diann \
  -d FragPipe_f374_diann/dataset_diann_example.xlsx
```

- run qc for 187 files

```
./prolfqua_qc.sh -s DIANN -i FragPipe_f374_diann \
  -d dataset_all_parallel.xlsx -o qc_dir_f374_diann
```

- generate DEA with factorial design

```
./prolfqua_dea.sh -s DIANN -i FragPipe_f374_diann \
  -d dataset_all_interaction_no_Subject.xlsx \
  -y config_vsn.yml -w f374_diann_with_interaction
```

```
resDS <- runtime_DEA("FragPipe_f374_diann/prolfqua_logMemUsage_dataset.log")
resQ <- runtime_DEA("FragPipe_f374_diann/prolfqua_logMemUsage_qc.log")
resD2 <- runtime_DEA("FragPipe_f374_diann/prolfqua_logMemUsage_dea.log")

timing <- data.frame(name = c("Dataset", "QC", "DEA1"),
  RAM_GB = c(resDS$maxGB, resQ$maxGB,resD2$maxGB),
  Time_min = c(resDS$maxTime, resQ$maxTime, resD2$maxTime))

knitr::kable(timing,caption = "f374 DIANN report tsv input.")
```

f374 DIANN report tsv input.

name	RAM_GB	Time_min
Dataset	67.31651	21.21667
QC	74.44155	67.38333
DEA1	67.45133	72.95000

DIANN report.tsv with 561 samples.

This dataset was create by duplicating the dataset with 187 samples.

- generate dataset to annotate samples

```
./prolfqua_dataset.sh -s DIANN -i FragPipe_f561_diann \
  -d FragPipe_f561_diann/dataset_diann_example.xlsx
```

- generate qc report

```
./prolfqua_qc.sh -s DIANN -i FragPipe_f561_diann \
  -d dataset_all_parallel.xlsx -o qc_dir_f561_diann
```

- generate DEA with factorial design

```
./prolfqua_dea.sh -s DIANN -i FragPipe_f561_diann \
  -d dataset_all_interaction_no_Subject.xlsx \
  -y config_vsn.yml -w f561_diann_with_interaction
```

```
resDS <- runtime_DEA("FragPipe_f561_diann/prolfqua_logMemUsage_dataset.log")
resQ <- runtime_DEA("FragPipe_f561_diann/prolfqua_logMemUsage_qc.log")
resD2 <- runtime_DEA("FragPipe_f561_diann/prolfqua_logMemUsage_dea.log")

timing <- data.frame(name = c("Dataset", "QC", "DEA1"),
  RAM_GB = c(resDS$maxGB, resQ$maxGB,resD2$maxGB),
  Time_min = c(resDS$maxTime, resQ$maxTime, resD2$maxTime))

knitr::kable(timing,caption = "f561 DIANN report tsv input")
```

f561 DIANN report tsv input

name	RAM_GB	Time_min
Dataset	99.03707	30.05000
QC	107.13995	89.78333
DEA1	98.94452	93.73333

R version and session information

```
pander::pander(sessionInfo())
```

R version 4.4.2 (2024-10-31)

Platform: x86_64-pc-linux-gnu

locale: *LC_CTYPE=en_US.UTF-8*, *LC_NUMERIC=C*, *LC_TIME=en_US.UTF-8*, *LC_COLLATE=en_US.UTF-8*, *LC_MONETARY=en_US.UTF-8*, *LC_MESSAGES=en_US.UTF-8*, *LC_PAPER=en_US.UTF-8*, *LC_NAME=C*, *LC_ADDRESS=C*, *LC_TELEPHONE=C*, *LC_MEASUREMENT=en_US.UTF-8* and *LC_IDENTIFICATION=C*

attached base packages: *stats*, *graphics*, *grDevices*, *utils*, *datasets*, *methods* and *base*

loaded via a namespace (and not attached): *digest(v.0.6.37)*, *codetools(v.0.2-20)*, *lubridate(v.1.9.4)*, *fastmap(v.1.2.0)*, *xfun(v.0.49)*, *knitr(v.1.49)*, *htmltools(v.0.5.8.1)*,

timechange(v.0.3.0), *rmarkdown(v.2.29)*, *generics(v.0.1.3)*, *cli(v.3.6.3)*, *pander(v.0.6.5)*, *compiler(v.4.4.2)*, *tools(v.4.4.2)*, *evaluate(v.1.0.1)*, *Rcpp(v.1.0.13-1)*, *yaml(v.2.3.10)*, *rlang(v.1.1.4)* and *jsonlite(v.1.8.9)*

Supporting Material S3: Prolfquapp DEA SummarizedExperiment visualized with exploreDE

Contents

Visualizing DEA results with the *exploreDE* application **1**

Deploying and Running the *exploreDE* application **2**

Visualizing DEA results with the *exploreDE* application

We show here how the *prolfqua_dea* application's results are visualized using the *exploreDE* application (https://zenodo.org/doi/10.5281/zenodo.10026461). The *prolfqua_dea* application generates *SummarizedExperiment* objects, which are serialized into the *SummarizedExperiment.rds* file. These files can then be loaded into the *exploreDE* shiny application. In this example, we use the *exploreDE* installation at the *FunctionalGenomicsCenterZurich*.

- DEA analysis of 20 samples and a parallel group design
- DEA analysis with 187 samples and a factorial design

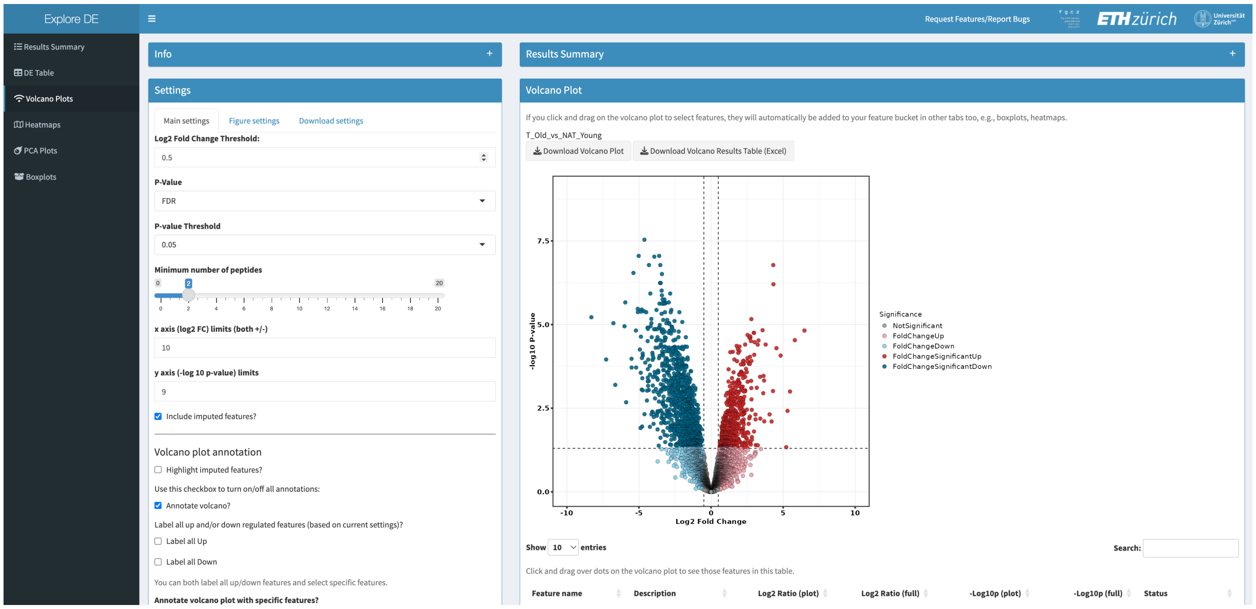

Screenshot of the exploreDE application.

For more details on how these analyses are generated, see *SupportingMaterialS2*.

Deploying and Running the *exploreDE* application

The *exploreDE* application is **independent** of the *prolfquapp* application. To visualize other datasets, you must host your *exploreDE* Shiny installation. This can be done by cloning the *exploreDE* Gitlab repository. Ensure that R, the Shiny package, and any required dependencies are correctly installed. A shiny application can be started from the R command line using `runApp()` from the *exploreDE* subfolder of the *rs_connect_apps* project. You must also edit the $server - initInputData.R$ file and provide the path to the *prolfquapp_dea* generated *SummarizedExperiment.rds* file at line 26. You can find the latest version of the *exploreDE* application at (https://gitlab.bfabric.org/hubert/rs_connect_apps)[https://gitlab.bfabric.org/hubert/rs_connect_apps].
